# Disentangling predictive processing in the brain: a meta-analytic study in favour of a predictive network

**DOI:** 10.1038/s41598-021-95603-5

**Published:** 2021-08-10

**Authors:** Linda Ficco, Lorenzo Mancuso, Jordi Manuello, Alessia Teneggi, Donato Liloia, Sergio Duca, Tommaso Costa, Gyula Zoltán Kovacs, Franco Cauda

**Affiliations:** 1 Focuslab, Department of Psychology, University of Turin, Turin, Italy; 2 GCS-fMRI, Koelliker Hospital and Department of Psychology, University of Turin, Turin, Italy; 3 Department of Biological Psychology and Cognitive Neuroscience, Institute for Psychology, Friedrich-Schiller University of Jena, Jena, Germany; 4 Department for General Psychology and Cognitive Neuroscience, Friedrich Schiller University Jena, Am Steiger 3/Haus 1, 07743 Jena, Germany

**Keywords:** Neuroscience, Psychology

## Abstract

According to the predictive coding (PC) theory, the brain is constantly engaged in predicting its upcoming states and refining these predictions through error signals. Despite extensive research investigating the neural bases of this theory, to date no previous study has systematically attempted to define the neural mechanisms of predictive coding across studies and sensory channels, focussing on functional connectivity. In this study, we employ a coordinate-based meta-analytical approach to address this issue. We first use the Activation Likelihood Estimation (ALE) algorithm to detect spatial convergence across studies, related to prediction error and encoding. Overall, our ALE results suggest the ultimate role of the left inferior frontal gyrus and left insula in both processes. Moreover, we employ a meta-analytic connectivity method (Seed-Voxel Correlations Consensus). This technique reveals a large, bilateral *predictive network*, which resembles large-scale networks involved in task-driven attention and execution. In sum, we find that: (i) predictive processing seems to occur more in certain brain regions than others, when considering different sensory modalities at a time; (ii) there is no evidence, at the network level, for a distinction between error and prediction processing.

## Introduction

According to the theory of predictive coding (PC)^1–5^, our brain constantly attempts to model the probability of its own future states, with the goal of minimizing uncertainty^4^. More specifically, the brain is considered a hierarchically organized system where, at each level of processing, higher layers try to predict the latent causes of the sensory input coming from lower layers^6,7^. Thus, neurons at higher levels encode predictions about the upcoming signal, which is continuously compared with the effective signal received from lower levels. Through this comparison, the brain either reinforces existing predictions or it updates them, if these do not match the incoming signal^8^. When predictions are violated, a prediction error signal^5,9,10^ is fed back to the neurons encoding predictions. These recursive loops of predictions and error signals ultimately allow the individual to maintain up-to-date representations about its own internal states^11^ and the surrounding external stimuli. Over the past two decades, PC theory has received extensive support from a vast range of theoretical and experimental studies, both in relation to primary sensory processes^5,12–14^ and higher level cognitive processes^15,16^, such as decision making and naturalistic speech comprehension^14,17,18^. Moreover, evidence has been obtained with a variety of methods, mostly with functional magnetic resonance imaging (fMRI), but also electroencephalography^19–21^, computational simulations^22^, transcranial magnetic stimulation^23^, and physiological recordings of single neurons (for a review, see^24^).

Since 1999, when Rao and Ballard published their seminal simulation work on predictive coding in the visual cortex^5^, there has been a proliferation of attempts to implement PC in the human brain. Initially, it was argued that predictive processing occurs at the cellular level^25^, where the activity of neural populations is modulated by higher-order predictions and units signalling precision of those predictions. According to Bastos and colleagues^26^, PC is a typical property of the human cerebral neocortex because its structure suits a hierarchical signal exchange between cortical layers. In particular, error signals seem to be computed in the granular layers (especially layer IV), while predictions would be encoded in layers II and III^26^. These mechanisms have been identified in a large set of brain areas, including the primary sensory and motor cortices, motor association cortices, dorsal and ventral prefrontal cortices, parietal cortex, anterior cingulate cortex, insula, hippocampus, amygdala, basal ganglia, thalamus, hypothalamus, cerebellum and the superior colliculus^27,28^. However, in all these regions, neuronal units producing error signals and those encoding predictions of future states are functionally separated^29^. This separation has been found empirically through computational modelling, where neurons encoding predictions were found to be located in cortical layers II/III and prediction error neurons in layer IV^22^. Based on these considerations and a previous study^30^, in this work we consider the functions of prediction encoding and violation in two separate conditions.

Current views conceive brain functions as a product of the co-activity of distributed brain networks^31,32^. This shift has also influenced implementations of PC in the brain. Specifically, while earlier formulations ground PC mechanisms in different layers of the human cortex, more recent models of PC attribute functions of error computation and prediction encoding to discrete brain regions and their long-range interconnections^33–35^. However, such models describe PC-related networks in isolated domains, such as face processing^33^. Thus, the question of whether the same network structure exists for the encoding of predictions and transmission of error signal across sensory modalities, domains and experimental paradigms remains open. To our knowledge, no previous study has addressed the existence of a predictive network with meta-analytic functional connectivity methods. In addition, compared to previous studies (e.g.^30^), we aim to include a wider variety of experimental paradigms and sensory modalities. First, we performed a coordinate-based meta-analysis, then we calculated the functional connectivity of the regions which were activated in the original studies. We formulated the following hypotheses:

- At least some of the regions involved in predictive activity might be functionally connected, revealing a spatially defined network. Moreover, given the dense interchange of prediction and error signals in the human cortex^26,29^, and the heterogeneous nature of our datasets, our network might involve mostly higher order regions.
- As regards the brain areas generally involved in predictive processing, we might only partially replicate the results from a recent meta-analysis^30^, principally due to the diversity of selection criteria. While Siman-Tov and colleagues^30^ include studies pertaining to three specific domains, we include a wider range of effects and sensory modalities.
- As for the areas involved in prediction error computation, a recent meta-analysis^9^ highlighted the role of striatum, insula, thalamus and fronto-medial structures, while others^36^ reported other regions (the bilateral ventral striatum, the thalamus, the left frontal operculum, the left caudate and the left IFG). We therefore aimed to verify whether, with different selection and categorization criteria, these results on prediction error computation could be replicated.

## Results

### Selection of studies

**Figure 1:**
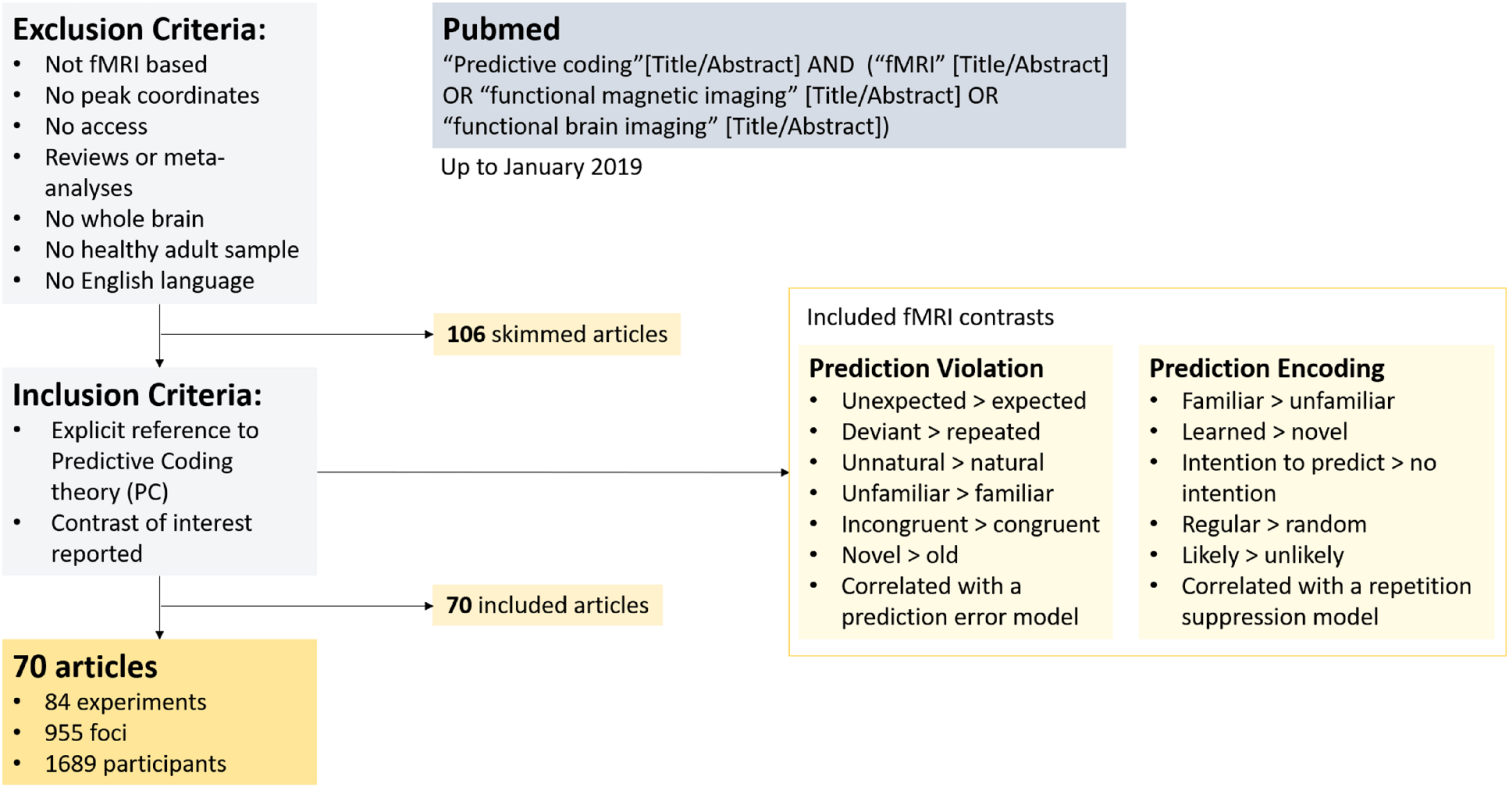
Flowchart representing the process of search and selection of potentially eligible articles for the meta-analysis and the SVC Consensus.

Following the criteria (a–f) described in “Selection of studies”, 106 articles were collected (see Fig. 1). Data from these articles were classified in a table specifying the study identification code, year of publication, first author’s last name, title, scientific journal, number of experimental subjects, experimental task, sensory modality investigated, experimental contrast, type of stimuli. A further selection based on criteria (g) and (h) led to 70 articles. All the peak coordinates listed for the experimental contrast, which were classified as Prediction Encoding or Prediction Violation, were reported in a separate table. The classification of each reported contrast in the two conditions can be found in the Supplementary Tables S1 and S2. When necessary, we converted the peak coordinates to the Montreal Neurological Institute (MNI) space, using the icbm_spm2tal transform on GingerALE^37,38^ (http://www.brainmap.org/icbm2tal/).

### Activation likelihood estimation

**Figure 2:**
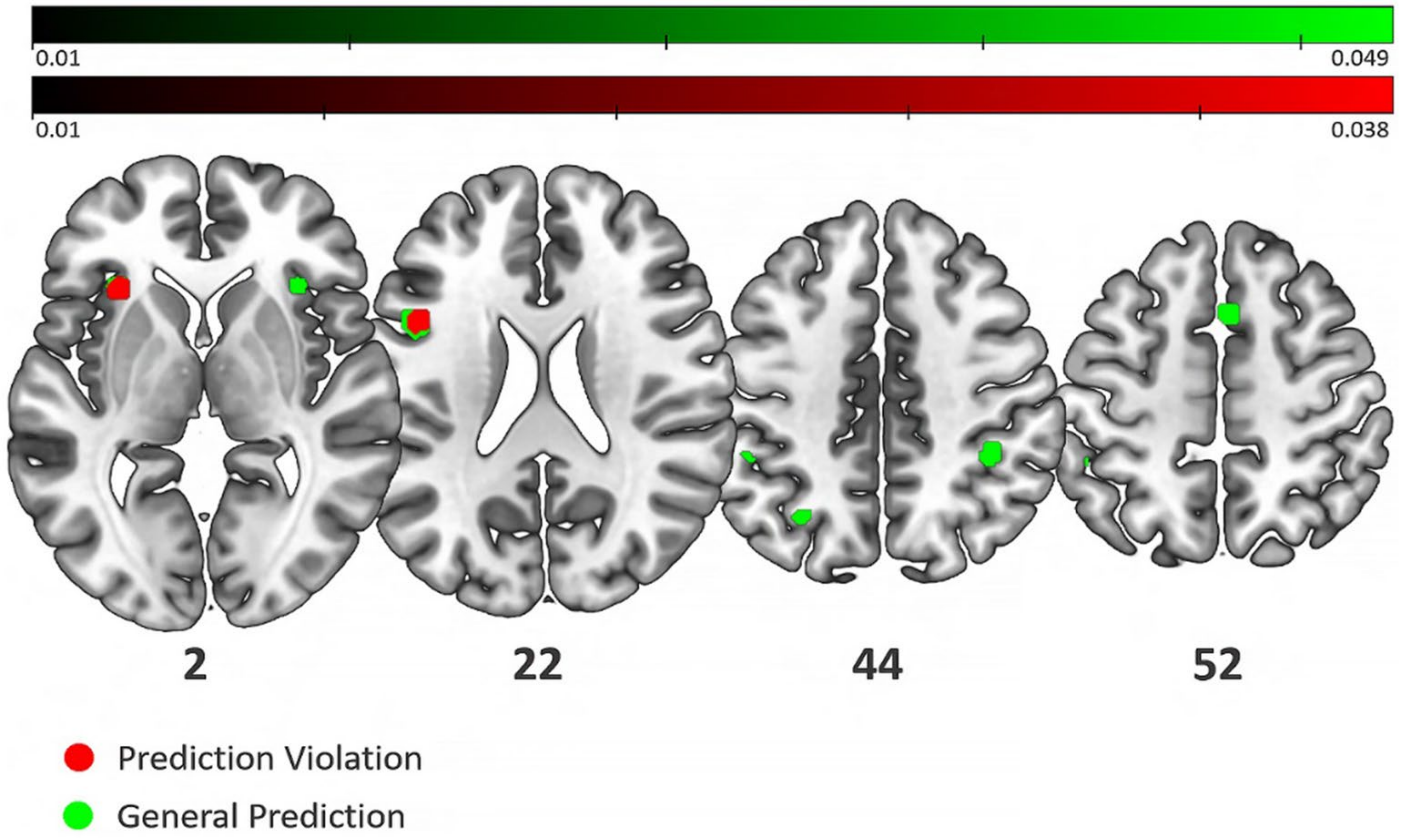
Activation Likelihood Estimation results at a FWE-corrected voxel-level threshold (p < 0.05). Green: condition of General Prediction; Red: condition of Prediction Violation. Two clusters are in common between the two conditions, one in the left anterior insula/claustrum and the other in the left inferior frontal gyrus/precentral gyrus. The General Prediction condition also shows clusters in the right insula, right and left inferior parietal lobule, one in the cuneus and one in the right middle frontal gyrus.

A detailed explanation of how each contrast was classified as reflecting the effect of prediction encoding and violation is reported in “Selection of studies”. As a first step, we performed ALE meta-analyses singularly on the two main conditions: Prediction Violation (45 experiments, 511 foci and 939 subjects) and Prediction Encoding (39 experiments, 444 foci, 750 subjects). Afterwards, we run an ALE meta-analysis on a unified dataset, derived by pooling the coordinates relative to the two conditions (70 experiments, 930 foci of activation and 1419 participants). We refer to these analyses as General Prediction. Figure 2 shows the results of the ALE analyses at FWE, p < 0.05. Further details of the ALE results are reported in Table 1.

**Table 1:** Activation likelihood estimation (ALE) results.

Condition	MNI coordinates (x, y, z)	Volume (mm^3^)	Maximum ALE value	Z score	P value	Anatomical location (Brodmann area)
General Prediction*	− 46, 10, 24	1088	0.049	6.78	6.11e−12	Left inferior frontal gyrus (BA 9)
	− 30, 24, 2	608	0.043	6.10	5.23e−10	Left insula (BA 13)
	4, 14, 50	224	0.037	5.39	3.59e−8	Right superior frontal gyrus (BA 6)
	34, 24, 2	200	0.034	5.09	1.83e−7	Right insula (BA 13)
	− 28, − 64, 46	200	0.034	5.13	1.44e−7	Left precuneus (BA 19)
Prediction Encoding**	36, − 60, 50	832	0.021	4.47	3.96e−6	Right superior parietal lobule (BA 7)
	− 50, − 44, 52	608	0.021	4.38	5.82e−6	Left inferior parietal lobule (BA 40)
	4, 12, 50	584	0.026	4.98	3.19e−7	Right Superior frontal gyrus (BA 6)
	− 38, − 80, − 14	528	0.023	4.67	1.49e−6	Left fusiform gyrus (BA 19)
	48, − 64, − 8	464	0.018	3.98	3.44e− 5	Right fusiform gyrus (BA 19)
	− 46, 8, 26	440	0.023	4.61	2.03e−6	Left inferior frontal gyrus (BA 9)
	48, − 44, 46	328	0.020	4.19	1.41e−5	Right inferior parietal lobule (BA 40)
	28, − 6, − 20	312	0.021	4.35	6.70e−6	Right amygdala
	42, 22, 32	288	0.019	4.14	1.76e−5	Right middle frontal gyrus (BA 9)
	− 28, − 6, − 20	224	0.020	4.25	1.05e−5	Left amygdala
	− 26, − 64, 46	224	0.019	3.98	3.40e−5	Left precuneus (BA 7)
	46, 18, 6	192	0.021	4.29	8.81e−6	Right insula (BA 13)
	20, − 100, 6	136	0.018	3.82	6.80e−5	Right cuneus (BA 17)
	66, − 22, 6	120	0.017	3.79	7.41e−5	Right superior temporal gyrus (BA 42)
Prediction Violation*	− 46, 10, 24	616	0.033	5.59	1.12e−8	Left inferior frontal gyrus (BA 9)
	− 30, 24, 0	608	0.038	6.19	2.94Ee−10	Left insula (BA 13)

Convergent findings of brain activity related to predictive coding conditions. *Significant activations are set at voxel-level p < 0.05 with the Family-Wise Error (FWE) correction. **Significant activations are set at p < 0.0005 uncorrected for multiple comparisons.

#### Prediction violation

Two significant clusters were related to the violation of predictions (Fig. 2, red color). The larger cluster included the left inferior frontal gyrus, while a smaller cluster was found over the left anterior insular cortex, partially overlapping with the claustrum.

#### Prediction encoding

No significant cluster was found for Prediction Encoding at the typically applied, conservative threshold of FWE, p < 0.05. Lowering the threshold to FDR, p < 0.01 still did not produce any significant convergence. However, at an exploratory level, we report the results obtained at a more liberal threshold (*Uncorrected, p* < 0.0005). At this threshold, fourteen clusters emerged. These included the right superior and left inferior parietal lobules, the right superior, right middle and left inferior frontal gyri, the bilateral fusiform gyri and the right amygdala, and a few clusters with a size inferior to 200 mm^3^ (including the left amygdala, the left precuneus and the right cuneus, the right insula and the right superior temporal gyrus).

#### General prediction

Overall, the ALE analysis of the whole dataset returned a set of cortical regions in the frontal and parietal lobes (Fig. 2, green). These include the left inferior frontal gyrus, the insulae bilaterally, the right superior frontal gyrus, the bilateral inferior parietal lobules, and the left precuneus.

### Seed-voxel correlations consensus

**Figure 3:**
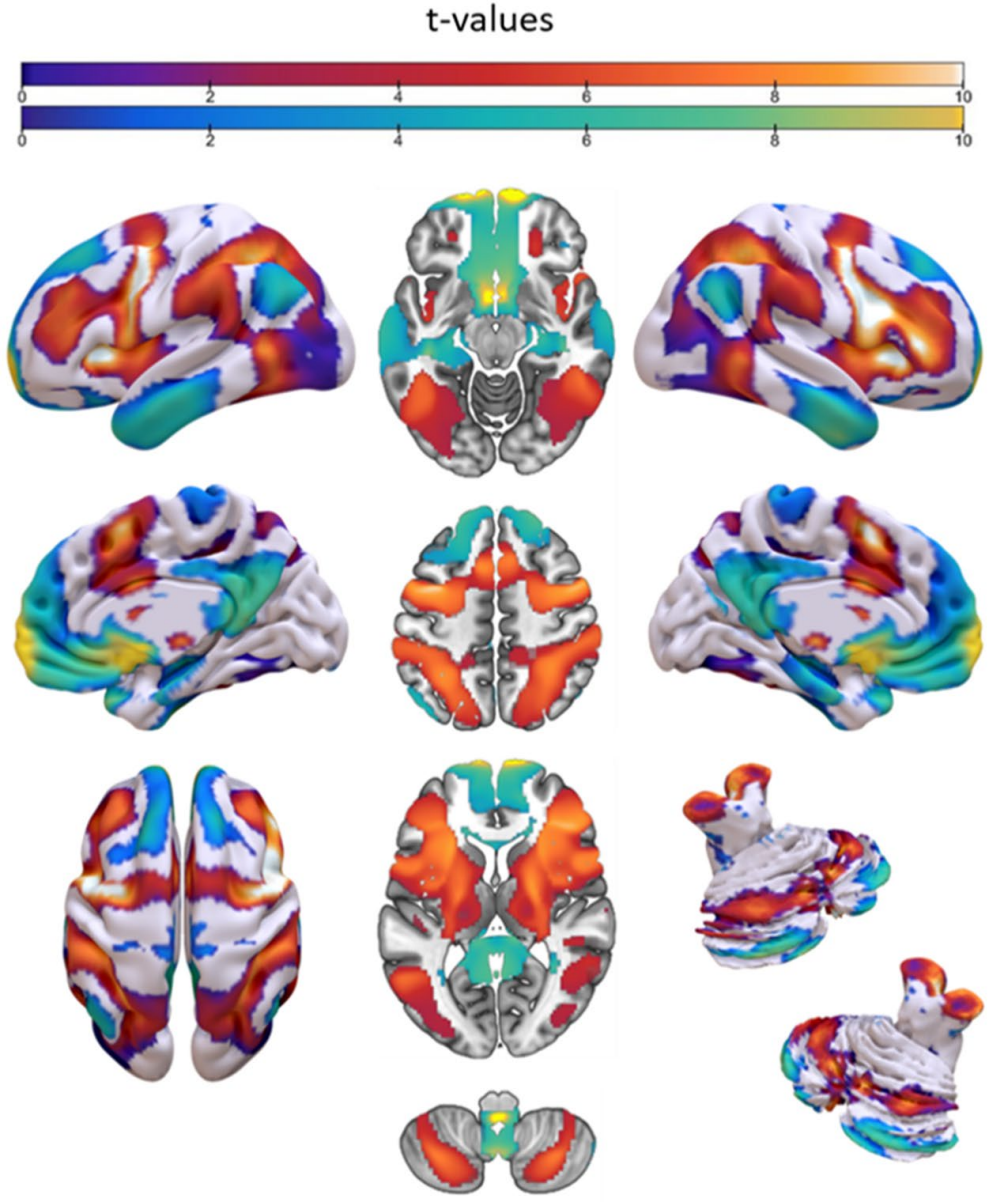
Surface, medial and cerebellar mapping of the SVC Consensus analysis revealing the “predictive network” for the condition of General Prediction. Note that this and the Prediction Violation areas overlap entirely. The network that shows the Prediction Encoding areas is presented in the Supplementary Materials (Fig. S2). Warm colors represent positive t-values (range: 4.37–14.17), cold colors are for negative t-values (range: 4.37–19.61), shown in arbitrary units.

This technique highlights the regions showing correlated activity with those that were active during the tasks tapping into predictive processing (see “Seed-voxel correlations Consensus”). Overall, the results from all the three conditions are remarkably similar, thus we focus on the results of the General Prediction condition (${SIM}_{General/Encoding}=0.78$; ${SIM}_{General/Violation}=0.93$; ${SIM}_{Encoding/Violation}=0.68$). Peaks are located in the left inferior frontal gyrus, the superior temporal gyrus bilaterally, the left thalamus, the left hippocampus and the left cerebellum. Significant voxels are shown in warm colors. The network emerging from negative correlations (cold colors) includes the right cerebellum (uvula), the left precentral gyrus and the post-central gyri bilaterally, and the right middle occipital gyrus (Fig. 3; see Table S2 for more details). Finally, the network relative to Encoding is substantially overlapping with that of the other two conditions, although the map of positive values appeared to be less extended. The major regions of differential connectivity between this and the map of Prediction Encoding include the left insula, the left middle frontal gyrus, the left anterior cingulate gyrus and the inferior frontal gyrus bilaterally (Fig. S3).

**Figure 4:**
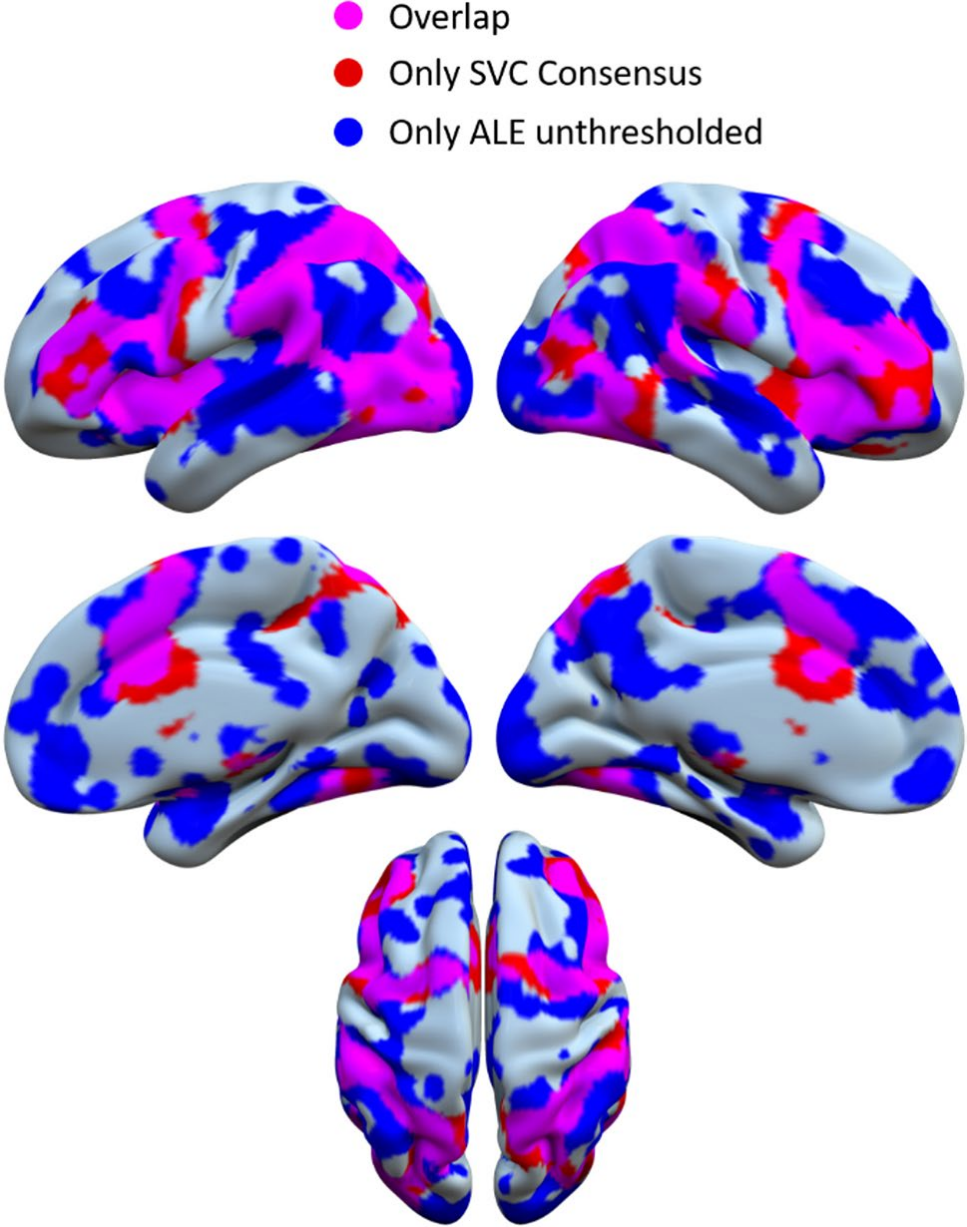
Surface map showing the overlap between the results of the unthresholded ALE (blue) and the SVC Consensus (red) analyses, for General Prediction. The overlapping regions are presented in purple.

Although the SVC Consensus maps indicate regions that are significantly connected to the activation foci reported in the literature, the relation between these foci and such maps needs to be clarified. In fact, on one hand it is possible that only a few foci are responsible for the connectivity maps. On the other hand, these maps might show areas that are not reported in the literature (thus are not primarily considered to be involved in predictive processing), but are systematically connected to the predictive regions, possibly providing input or output to them. To investigate the relation between the foci and their connectivity, we overlapped the SVC Consensus of the General Prediction condition to the corresponding unthresholded ALE map. Here, the unthresholded map can be seen as an indicator of all the activated regions in the literature. We found out that there is a substantial overlap between the two maps (Fig. 4), suggesting that the activated areas tend to be interconnected and to form a coherent functional network ($SIM = 0.56$). Lastly, to exclude the possibility of bias due to local connectivity, the SVC Consensus analysis was repeated excluding proximal connections between close areas from the SVC maps. The resulting maps were extremely similar to those obtained with the original SVC Consensus maps, suggesting that activation foci are connected not only to the spatially closer areas, but also to the more distal ones (Fig. S1).

### Fail-safe technique

**Figure 5:**
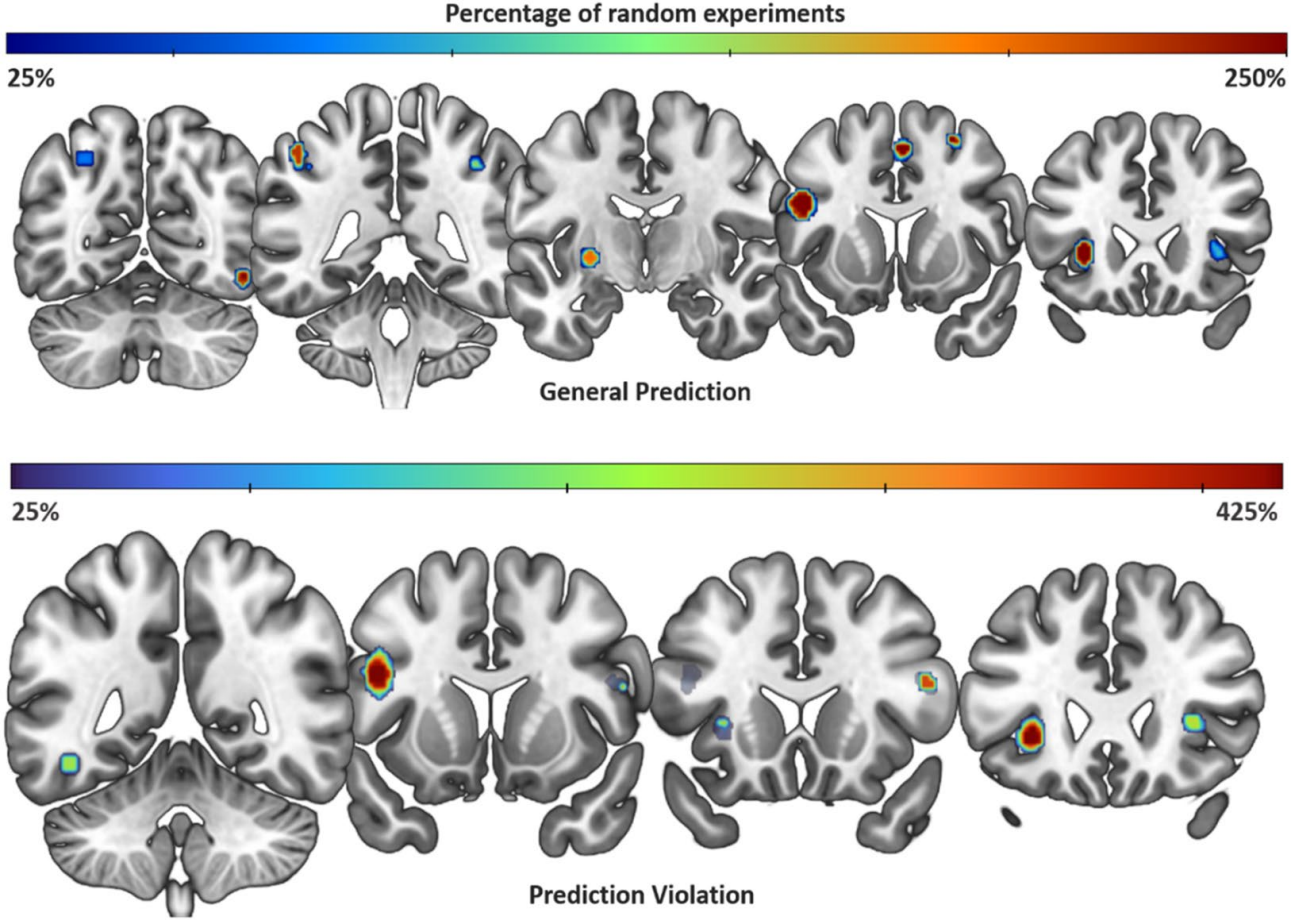
Coronal sections showing the results of the fail-safe analysis. Upper row: General Prediction; lower row: Prediction Violation. The color scales represent gradually increasing added random data in percentage. The warmer the color of the pixels, the more random noise (representing potentially unpublished results) is tolerated.

To test the impact of potential selection bias, we performed a fail-safe analysis^39^ (“Fail-safe technique”). In the General Prediction condition, the analysis shows that at least one of the clusters remains significant up to the introduction of 250% random data, suggesting their robustness against selection bias (Fig. 5). The analysis of the Prediction Violation dataset suggests an even greater robustness (the clusters remain significant up to the inclusion of 425% random data). In general, both fail-safe tests suggest the robustness to bias of the two clusters that are in common for the two conditions, i.e. the left IFG/precentral gyrus and the left insula/claustrum.

### Leave-N-out

**Figure 6:**
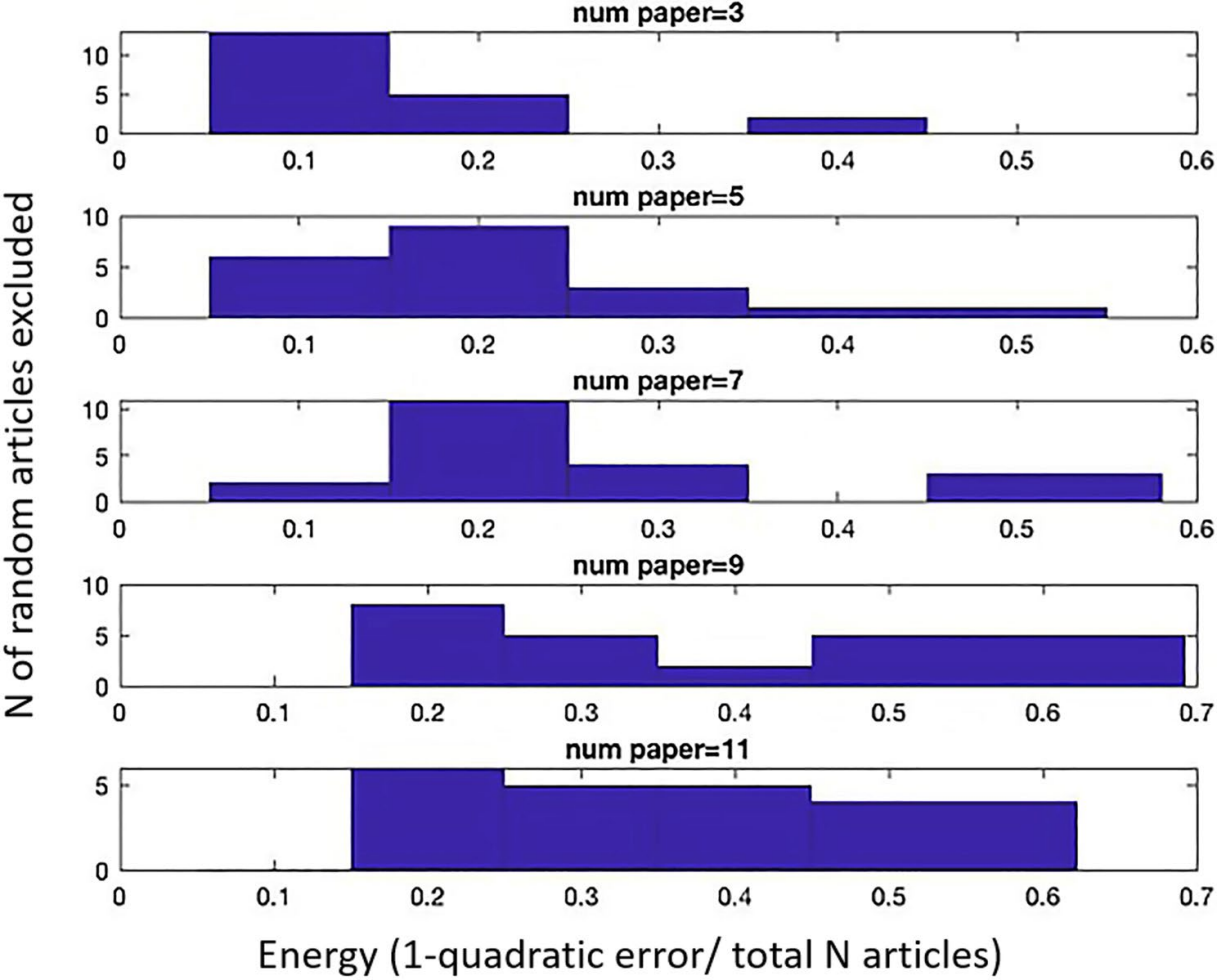
Diagram relative to the results of the leave-N-out analysis on the articles included in the Prediction Encoding condition. In each section, the y-axis shows the number of articles, and the x-axis shows the energy (1-quadratic error/total N experiments). To calculate the standard error, each run with removal and reinsertion of ‘N’ articles was repeated 10 times. Since removing up to 7 articles at a time (18% or the total dataset) still did not introduce important changes in the distribution of energy, we conclude that the dataset is homogeneous. See Section 4.5 for more details.

This analysis tests the heterogeneity of a dataset, or whether all the studies in a dataset contribute to the results similarly (Section 4.5). It was performed on the condition of Prediction Encoding due to the lack of convergence. Figure 6 shows the results from the leave-N-out analysis. The y axis indicates the number of papers, while the x axis the energy (1-quadratic error/total N experiments). The diagrams show the distribution of energy obtained by removing 3, 5, 7, 9 and 11 articles at each run separately. When removing less than 7 random articles at a time, no important changes are visible in the distribution. Since 7/39 (removed articles/total) equals to 18% of the included experiments, this suggests that the condition of Prediction Encoding is mostly homogeneous. Thus, it is unlikely that the absence of convergence is due to the heterogeneity in the experiments. Instead, it may be due to the spatial distributedness of the activation coordinates per se.

## Discussion

In this study, we partially replicate convergence results from previous meta-analyses. In addition, we provide evidence for the existence of a network involved in PC across sensory modalities and a variety of tasks. This spatially extended and bilateral network overlaps with known large-scale networks supporting attention and task execution. Finally, although the separation between error, weighting and encoding units is supported by our ALE results and previous work, our findings suggest that the regions that are engaged during prediction violation and encoding tend to be functionally connected with the same network.

The ALE results show convergence across tasks targeting predictive processing in a set of cortical regions, in both the Violation and the General condition. However, we did not find convergence in the Encoding condition, even at more liberal thresholds. As suggested by the results of the leave-N-out analysis, this spatial heterogeneity does not seem to be due to the disproportionate contribution of few studies, but rather to the large variability in the localization of foci. A qualitative inspection further indicates that this spatial distributedness is not due to a larger heterogeneity in the selected tasks either. Rather, it likely reflects a wider range of effects, due to our definition of prediction encoding. In fact, while the Violation condition mainly includes effects of surprise and stimulus randomness, that of Encoding reflects effects of item repetition, habituation/adaptation, belief updating, memory, high probability and, in some cases, an explicit effort to predict upcoming stimuli. However, we cannot exclude that the spatial heterogeneity is an intrinsic property of the process itself. For instance, it was found that the effect of belief updating, one of the effects we included in the Encoding dataset, did not replicate between different methods^40^. If other effects suffered from the same issue, this could perhaps explain their wide spatial distributedness. Another potential reason for the lack of convergence is that one of the typical effects we included is that of repetition suppression, which is defined as a decrease in brain activity in task-related areas^41^. Therefore, this effect is probably at least partially more task-dependent in nature, thus leading to reduced convergence. It is challenging to compare this result to that by Siman-Tov et al., since only results from a subtraction between violation and formation of prediction are reported^30^. Nevertheless, we compared a meta-analysis on visual repetition suppression^42^ with the results of a subgroup analysis on a subset of studies employing visual stimuli from the Encoding condition. While Kim et al. report a network including visual cortices, frontal and parietal regions and the caudate, we again obtained no significant convergence, at the same threshold. This further strengthens the idea that the effects included in the Prediction Encoding condition show strong task-dependency. Although we preferred not to compare such effects systematically due to low power, future meta-analyses may quantitatively compare different aspects of prediction encoding.

One expected site of convergence that we have not found is the cerebellum, especially when analysing the violations of predictions. In fact, this structure has been reported in previous works as an important hub processing the comparison between an internal model and the current sensory input, and as a region that supports procedural and perceptual learning mechanisms^43–45^. However, there are two main reasons why few neuroimaging meta-analyses are able to detect convergence in the cerebellum^46^. First, there could be technical difficulties associated with the detection of BOLD signal from the cerebellum, principally dependent on experiments targeting climbing fibres, which are poorly coupled to this signal^46^. Second, some experimental paradigms tend to promote rapid habituation in this area, which in turn produces lower neural responses^46^.

Overall, our analyses of the Prediction Violation condition confirm previous findings^9,30,36^ regarding the insula and the IFG, while the involvement of others, such as the striatum and the thalamus, are not replicated. Both the insula and the IFG have been related to the violation of predictions in previous works. In particular, the anterior insulae have been related to the processing of bodily sensations and awareness of subjective feelings^47^. Moreover, insular regions compute prediction error signals, especially in the interoceptive modality^35,48,49^. Considering that only a small number of included studies explicitly targeted bodily sensations, this finding deserves special attention. A tentative interpretation is that, regardless of the nature of the specific expectations that are violated in each task, these tend to produce an error broadly related to the self^50^. The insular cluster also extends to include the left claustrum, which produces prediction error signals in Pavlovian classical conditioning paradigms^51^. Notably, these involve a component of automatic learning, which probably most of the tasks we included contain, to some degree. The second cluster was located in the IFG, which is involved in risk aversion^52^, and in detecting a mismatch between expectations and decisions^53^. Recent studies indicate that this region plays a role in the violation of expectations. For instance, a correlation was found between both IFG and insula activity with a prediction error model during bi-stable perception, which is a paradigm inducing strong violations of visual expectations^54^. Moreover, ERP components in both which are associated with surprise were larger than those related to belief updating the right IFG and the bilateral insulae^55^. Lastly, intrinsic connectivity between the IFG and the insula was predicted by the degree of intolerance of uncertainty, which further indicates their sensitivity to error signals^56^. Together, these findings and our results suggest that the insula and the IFG, and their connectivity during prediction violation across modalities, are a worthy avenue for further research.

The General Prediction condition was designed to tap into the general effects of predictive processing, that arise from the mere fact of performing a task eliciting predictions or prediction errors. Thus, we expected the brain regions emerging here to be related either to one or the other process, or to both. First, convergence was found in two larger clusters, one in the inferior frontal gyrus/precentral gyrus, and the other bilaterally in the insula. Strikingly, both regions also emerged in the meta-analysis of Siman-Tov et al., who similarly pooled together the effects of prediction encoding and violation^30^. This strengthens the plausibility of this result in other domains than those of music, action and language perception. Further supporting the double role of both the IFG and the insula in PC, activity in these areas represents the building of an expectation, analysing the conjunction across somatosensory, visual and auditory stimulus modality^57^. However, evidence about the IFG is somewhat more mixed. In fact, its activity does not always seem to depend on the predictability of a situation^58^. Moreover, whereas some authors describe it as an area involved in the processing of “expectancy input”^59^, others report increased activity in the IFG when the stimulus probability is low, leading to larger prediction error signal^60^. As regards the anterior insulae, notably these are an important hub of the salience network^61^. It is plausible that this hub is more activated in surprising situations driving attention^62^, which also involve an increased gain in error signal computation (the relationship between predictive and attentional processes is extensively discussed in the following paragraphs). Lastly, the role of precuneus in the General Prediction condition is more difficult to relate to the existing literature. In general, it is involved in self-related cognition, episodic memory and mental imagery^63^. Interestingly, this region was responsive to deviant stimuli even during sleep^64^, which may indicate a selective sensitivity to prediction error during different states of consciousness. However, belief updating modulates activity in this region as well^40^. Overall, these studies support our findings, and suggest that the IFG and the insula might be involved in both the encoding and the violation of predictions. More evidence on prediction violation than encoding exists in both cases, and the sensitivity of the IFG (and the less discussed precuneus) to stimulus probability may more strongly depend on specific task characteristics.

The SVC Consensus analysis was conducted to highlight the brain regions that tend to provide input or output to those involved in prediction violation and encoding. Since the resulting network is largely similar between conditions, we focus on the General Prediction condition.

An important aspect is that the maps relative to the Prediction Violation and Prediction Encoding conditions are highly similar ($SIM=0.68$, Fig. S2). This means that the regions involved in prediction cross-modally tend to exchange information with the same, broad set of areas during task execution. This finding is surprising for several reasons. First, as previously discussed, the Prediction Encoding condition reflects a greater diversity of effects than that of Violation. Second, a study examining functional connectivity during temporal and spatial predictions found that prediction violation and fulfilment modulate connectivity in distinct networks^65^. Lastly, this finding seems to contradict the fact that prediction violation and encoding are functionally separated^6,26,29^. Our ALE results further support this functional separation. Notably, all the regions differentially connected during the violation of predictions (Prediction Violation > Prediction Encoding) have been reported in a meta-analysis on prediction error during reinforcement learning^51^, an experimental paradigm designed to provoke a strong error signal. Still, despite minor differences between these two maps (Figs. S2 and S3), our results strongly suggest that the same network supports both functions.

A key feature of the network that we obtained in all conditions is its remarkable similarity to the so-called task-positive network (TPN^66^). The TPN is a set of areas involved in task execution, and is usually divided into three large-scale brain networks related to salience processing^61^ and the dorsal and the ventral attentional networks^67,68^.

The fact that the regions which are more involved in prediction are also part of attentional networks replicates the findings by Siman-Tov and colleagues^30^ and is of key theoretical importance. An increasing body of research considers prediction and attention as dissociable but strongly interdependent processes (for empirical evidences, see^69–71^; for further readings see^72–75^). Attention adjusts the computational weight (precision) of prediction error units via synaptic gain enhancement^7,76,77^, leading to increased error signals. While attention enhances the processing of relevant information and regulates the overall cortical responsiveness^78–80^, prediction allows the brain to take prior information into account^81^. Moreover, prediction “anchors” attentional processing, meaning that computing predictions is necessary to subsequent attention orientation^71^. Thus, the overlap between our map and the TPN could be interpreted in several ways. First, despite being distinct processes^82–85^, they may share a common neural territory. Considering that this network relies on the original coordinates of brain activations during task performance, it is clearly possible that both attention to the actual stimuli and the prediction of future stimuli were working simultaneously. Second, when multiple modalities are considered together, the activity of prediction and error computation might specifically involve attentional networks more than other brain regions. This has never been observed before because, for obvious practical reasons, only a limited set of modalities are investigated at a time, often in a rather constrained experimental setting. Finally, a third possibility is that the TPN emerged from our analyses merely for the effect of participants’ engagement in any attention-demanding task, and the selected contrasts do not reflect predictive processing at all. It is difficult to rule this possibility out completely, as we did not analyse an arguably non-predictive neutral control condition^86^. However, as we performed a careful selection of neuroimaging contrasts targeting the effects of interest, the overlap with the canonical attentional networks might imply some relationship between the two processes.

Finally, our predictive network appears to be negatively correlated with the default mode network (DMN; for example, see Fig. 3, negative values). Activity patterns of the DMN and those of the TPN are anticorrelated^87^, and possibly involved in different forms of cognition. In particular, the DMN is typically reported to be more activated during rest and mind-wandering^88^. Considering recent work suggesting that this network creates and updates internal predictive models about the self^89^, and that it is engaged when stimuli are temporally predictable^90^, the lack of connectivity within its key hubs is rather unexpected, especially in the Encoding condition (cf.^40^). Moreover, since the DMN is located at the extreme end of a continuum of integration and hetero-modal functioning within the human connectome^91,92^, it is even more surprising that it did not result from our functionally heterogeneous dataset. A possible reason for its absence could be that, under the hypothesis that the DMN is responsible for the integration of predictions in prior internal models—acting in a sort of “autopilot mode”^93,94^—almost no included experimental paradigm tested this kind of automatic activity. High temporal resolution techniques, computational, and meta-analytic approaches to functional neuroimaging data can be valuable tools to investigate the role of the DMN in predictive processing in the future.

While we have highlighted the theoretical relevance of our results in the previous paragraphs, the finding of a predictive network could also be meaningful in the clinical context. Debilitating clinical conditions seem to stem from alterations in the production of prediction error signals (e.g. schizophrenia, anxiety^95–98^), hyper-rigidity of prior information or inflexible precision of prediction error (e.g. autism^99–101^). Since psychopathology tends to spread in the brain exploiting existing functional connectivity patterns^102–105^, and our network is thought to reflect the connectivity between regions involved in cross-modal prediction, investigating this network in different psychiatric samples should be fruitful.

One potential limitation of the current work is that the selection, classification and coding of the articles was conducted manually by one author only. However, the coded dataset was independently cross-checked by another author. Moreover, a section of notes was included in the database with the aim to make the interpretation and selection processes more transparent, as suggested by recent guidelines^106^. Another potential flaw is caused by the heterogeneity of the definition of “prediction” in the literature. In fact, the concepts of prediction, anticipation and expectation are often used interchangeably^107^ and how they are operationalized in each study can potentially lead to confusion with other processes^81,108^. Lastly, the strong presence of studies employing visual or audio-visual tasks may have also limited the validity of the current results (Table S4). However, the absence of early visual areas in both the ALE and the SVC Consensus results may suggest that the impact of this imbalance is nevertheless limited.

## Methods

### Selection of studies

We searched for publications (Pubmed, https://www.ncbi.nlm.nih.gov/pubmed/) up to January 2019. Articles were chosen using the keywords “predictive coding” AND “fMRI”, OR “functional magnetic imaging” OR “functional brain imaging” in the title or in the abstract of the articles. The decision to choose the only term “predictive coding” instead of a variety of related terms had two purposes: on one hand, to select only articles explicitly explaining their results under this framework; on the other hand, we did not include terms as “prediction error”, “expectation” or “Bayesian brain” so that articles describing the role of expectancy in psychology without referring to PC could be excluded.

In the initial selection stage, the following primary inclusion criteria were applied. We included studies:

- which employed fMRI;
- which provided the peak coordinates of significant activation in stereotactic brain space (MNI, TAL). In some cases, we included coordinates derived from model fit or correlations with model parameters reflecting the effect of interest. Peak coordinates reflecting functional connectivity were excluded;
- which were original experimental works. We excluded reviews or other meta-analytic studies;
- which reported whole-brain analysis for the contrasts of interest (i.e., contrasts which were based on a priori regions of interests (ROI analyses)  were excluded);
- which were based on a healthy adult sample;
- which were written in English.

Additional, more specific criteria for inclusion were the following:

(g) the articles had to explicitly support the PC theory, regardless of the sensory modality and the process investigated (i.e., only studies bringing evidence in favour of the framework were considered);
(h) the articles had to include experimental contrasts which reflect the violation of a prediction or the creation, updating or maintenance of a predictive internal model.

First, we collected the contrasts fulfilling these criteria from each experiment. Afterwards, we assigned the coordinates to two conditions. The first condition, that we label Prediction Violation, includes tasks where the expectations of participants are unfulfilled. This effect is reflected in contrasts comparing, for instance, a deviant condition with an expected one, a mismatch condition with a match one, or a random condition with a regular one. Other studies similarly investigated prediction violation, by fitting a statistical model designed to represent the same effect. In general, such experimental conditions might cause a sense of surprise and increase prediction error signals^9^. The second condition, labelled as Prediction Encoding, contains a variety of effects, reflecting the neural encoding of expectations about upcoming stimuli or the learning of their statistical regularities^109^. Typically, these included contrasts between a learned and an unfamiliar condition/event, or between an expected and an unexpected condition. However, others target the effect of consciously trying to predict future scenes, those of adaptation to repeated stimuli or the comparison between high and low probability conditions. Finally, in order to investigate the regions involved in prediction encoding and violation, we created a third condition (General Prediction), obtained by merging the datasets of the other two conditions.

Importantly, since it is frequent that coordinates related to the error or the encoding effect are reported in the same experiments from different contrasts, we did not classify the individual studies according to the two conditions, but the single contrasts reported in each study. In other words, the unit of analysis was each individual contrast, and not the study. Furthermore, this classification not only considered the performed task, but also the interpretation of the results that was provided by the authors. For instance, a reduced response in some brain areas, related to the repetition of stimuli, is often interpreted as repetition suppression (RS), hence this effect would be included in our Prediction Encoding category (for a review on how PC may explain RS, see^33^). Finally, although some authors pointed out that the effects of repetition and expectation suppression are different^86^, note that we merge both effects under the category of Prediction Encoding. Figure 1 shows a flowchart representing the described steps of the selection process. For a detailed list of the contrasts included in the study and the classification of their reflected effect, see Tables S1 and S2.

### Activation likelihood estimation

Activation Likelihood Estimation (ALE) is a meta-analytic technique which detects areas of convergence across peak coordinates of significant activations from functional neuroimaging studies^110–112^. In short, the current version of this algorithm models a Gaussian kernel for each activation peak, considering these as fixed-effects within each study, whereas studies are treated as random-effects. The width of the kernels accounts for between-subject and between-lab variations leading to spatial uncertainty, and it is based on the number of participants in each study^110^. Then, one Modelled Activation (MA) map is calculated for each study, unifying all the modelled peaks. Afterwards, a union of all the MA maps is performed, where each voxel contains an ALE score. In order to test for statistical significance, the algorithm compares the ALE scores of the obtained union map with a null-distribution of ALE scores, reflecting random spatial association between studies. Specifically, such distribution reflects the null hypothesis that the activation foci are randomly distributed throughout the brain, leading to convergence only by pure chance. Lastly, a correction for multiple comparisons is applied. We used GingerALE version 3.0 (http://www.brainmap.org/) to perform the above meta-analysis. The threshold for detecting significant convergence was set at voxel-level p < 0.05, with 1000 permutations with the family-wise error correction method (FWE), and the analyses were performed in the MNI152 coordinate space. Coordinates reported in TAL in the original study were converted using the icbm2tal transform prior to the ALE analysis^38^. When more contrasts in one study reflected the effect of interest in each condition (e.g. two contrasts reflecting different aspects of encoding), we merged their respective coordinates. This ensured that participants were not included in the analyses twice^106^.

### Seed-voxel correlations consensus

To investigate the connectivity patterns of the areas involved in predictive processing, we performed a Seed-Voxel Correlations (SVC) Consensus technique, adapted to functional data. This type of connectivity detects the brain regions providing input or output to those activated in the original studies. This technique was originally developed by Boes and colleagues^113^ to map the connectivity of brain lesions. It consists of overlapping several SVC maps, to verify if they tend to connect to a set of shared areas. The explicit aim of this method was to test if the spatial heterogeneity of brain lesion of a given deficit could be reduced to a common functional network^114^. Similarly, in the present work we aimed to evaluate the spatial variability of regions associated with predictive processing. Specifically, we hypothesized that the diverse activation foci which were reported in the literature might belong to a single brain network, and thus that they tend to be connected to each other. To do so, each peak that entered the ALE meta-analysis was searched in the Neurosynth resting-state database to obtain a functional connectivity map. This means that the voxel corresponding to a given coordinate of activation was taken as the seed of each SVC. Neurosynth (http://www.neurosynth.org) is an online database of functional meta-data which allows to easily obtain SVC maps calculated on the 1000 subjects of the Brain Genomics Superstruct Project (https://dataverse.harvard.edu/dataverse/GSP). Each SVC map was then considered as an individual subject in a second-level analysis. Then, the overlap of those maps was assessed by the means of a one-sample t-test on SPM12, (http://www.fil.ion.ucl.ac.uk/spm/software/spm12/) with a FWE-corrected threshold of p < 0.05. Both the positive and the negative contrasts were calculated, thus obtaining a map related to the shared positive correlations and one related to the negative ones. This analysis was carried out separately for the datasets of the three conditions (Violation, Encoding and General; for details see below). To facilitate the comparison between the conditions of Prediction Encoding and Violation, we perform a two-sample t-test on the two SVC Consensus maps (see Table S4, Fig. S3).

It should be noted that functional connectivity is partly influenced by physical closeness, so that spatially closer voxels tend to be connected more strongly^115^. As we worked with a large number of foci (930 in total), it may be argued that many of the seeds were close to each other, and thus their maps would display a high degree of overlap. Therefore, the SVC Consensus results could be biased because of the mere spatial closeness of a high number of peaks. To test this hypothesis, we repeated the SVC Consensus analysis of the General Prediction condition using only the distant connectivity of each seed, defined as all the voxels in each SVC map that were at least 14 mm^3^ far from the seed^115^. Hence, local connectivity of a seed is the volume within the radius of 14 mm around it. For this test, all the voxels around the seed in each of the SVC maps were set to 0, before recalculating the t-test.

### Fail-safe technique

The fail-safe technique allows the verification of the potential presence of selection bias in the dataset^39^. The so-called “file-drawer problem” indicates the possibility that there might have been studies that were not published because of null results or findings that were not consistent with the authors’ expectations. This problem affects both neuroimaging and standard effect-size meta-analyses^116^, so that unpublished studies are likely to contradict the results of the meta-analysis. The fail-safe analysis^39^ aims to detect selection biases, estimating how many of these studies would make the results of the meta-analysis non-significant. This is performed in two steps. First, we introduced in our datasets a number of maps with similar features to those included in the meta-analysis, but random foci location (spatial noise). These maps represent potentially unpublished neuroimaging studies. Second, we performed the ALE analysis on the original dataset multiple times, using the same threshold and correction settings of the main analysis, but each time with an increasing number of spatial noise maps. We performed this analysis on the conditions of Prediction Violation and General Prediction. Since no significant cluster emerged from the condition of Prediction Encoding at voxel-level FWE p < 0.05, we did not perform the fail-safe analysis in this case. The results of this procedure are generally considered robust if the convergence results are maintained after adding more than 200% of random data.

### Leave-N-out

The leave-N-out method is a cross-validation method to test the heterogeneity of a set of data. In the present study, it was employed on the dataset of the Prediction Encoding condition, to check if the reason behind the absence of significant clusters could be the particular impact of a study or group of studies in the meta-analysis. This method gives us the possibility to weight the contribution of each experiment (or group of experiments) and estimate the presence of “outliers”, i.e. studies which contribute to the result disproportionately, driving the outcome to a certain direction^117^. This analysis has been performed by calculating the ALE results each time omitting a growing number of ‘N’ experiments with reinsertion after each run. Each ‘leave-N-out’ iteration has been repeated 10 times to calculate the standard error. The calculation of 1—quadratic error divided by the total number of experiments that are removed from the analysis and reinserted at each step is a measure called *energy*. This value can be interpreted as a measure of the extent to which ALE results are affected by the removal of the articles, indicating how much the dataset is homogeneous. In our dataset, the level of energy that seemed to be associated with a change in its distribution is 0.6, thus the procedure at each leave-N stopped when this threshold was reached.

### Overlap between the unthresholded ALE map and the SVC Consensus map

In order to test the overall robustness of the network, obtained with the SVC Consensus technique, we created the overlap of the General Prediction Consensus and the unthresholded General ALE maps. An overlap between these maps shows brain regions that are functionally connected to each other. We tested the degree of overlap both by visually analysing the map and by calculating the Cosine Similarity Index (SIM). This is a widely used metric to assess the similarity between two vectors, which is unsensitive to their magnitude. It is calculated as the dot product of two vectors (in our case, the two maps) divided by the product of the two vectors' magnitudes. Then, the similarity between the two maps was calculated with the following formula:

$${SIM}_{AB}=\frac{{\sum }_{k=1}^{n}{A}_{i}*{B}_{i}}{\sqrt{{\sum }_{i=1}^{n}{A}_{i}^{2}}*\sqrt{{\sum }_{i=1}^{n}{B}_{i}^{2}}}$$

where ${A}_{i}$ and ${B}_{i}$ represent the two maps’ vectors, and *n* the number of voxels. This index ranges from 0 to 1, where 1 indicates, in our case, complete similarity. We calculate the SIM to compare the SVC Consensus maps between each other, to quantify their overall degree of overlap.

## Supplementary Information

Supplementary Information.

## Data Availability

The datasets generated during and/or analysed during the current study are available from the corresponding author on reasonable request.
