# Supplementary material for: Disentangling predictive processing in the brain: a meta-analytic study in favour of a predictive network
Source: Sci Rep. 2021 Aug 10;11:16258. doi: 10.1038/s41598-021-95603-5 (PMC8355157; doi:10.1038/s41598-021-95603-5)
Supplement: Supplementary file 1 — Supplementary Information. [file 41598_2021_95603_MOESM1_ESM.pdf]

Disentangling predictive processing in the brain: a study in favour of a predictive network

Linda Ficco[123], Lorenzo Mancuso[12], Jordi Manuello[12], Alessia Teneggi[12], Donato Liloia [12], Sergio Duca [2], Tommaso Costa[12], Gyula Kovács[3], Franco Cauda[12]

[1]Focuslab, Department of Psychology, University of Turin (Italy)
[2]GCS-fMRI, Koelliker Hospital and Department of Psychology, University of Turin, Turin, Italy
[3]Department of Biological Psychology and Cognitive Neuroscience, Institute for Psychology, Friedrich-Schiller University of Jena (Germany)

Supplementary Material

Supplementary contents:

Table S1. Methodological design of the fMRI experiments included in the meta-analysis…**pp. 2**

Table S2. Task modality and predictive phenomenon of the fMRI experiments included in the meta-analysis…**pp. 5**

Table S3. Seed-voxel correlations (SVC) Consensus results…**pp. 7**

Table S4. Two-sample t-test comparing the SVC Consensus maps in the conditions of Prediction Encoding and Prediciton Violation...**pp. 8**

Table S5. Proportions of task modalities in the three conditions...**pp. 9**

Figure S1. Overlap between the SVC Consensus maps calculated with and without voxels located around the seed in the SVC maps (local connectivity). ...**pp. 9**

Figure S2. SVC Consensus results for Prediction Violation and Prediction Encoding conditions…**pp. 10**

Figure S3. Two-sample t-test results comparing the maps of the Predition Violation and the Prediction Encoding condition…**pp. 10**

Supplementary References. Published articles included in the meta-analysis…**pp. 11**

Table S1. Methodological design of fMRI experiments included in the meta-analysis.
ID: reference artile; N: number of subjects.

ID	First author	Year	N	Type of task	Type of contrast
1	Allen	2016	30	roving somatosensory oddball + counting	deviant > standard
2	Andics	2013	18	volume change detection	i) alternation > repetition; ii) repetition > alternartion
3	Barascud	2015	16	listening + response to transitions	regular > random
4	Bartels	2007	8	passive observation	residual > motion-induced changes
5	Blank	2013	19	visual speech recognition	mismatch > match
6	Blank	2018	24	forced choice task	mismatch “different” percept > match “same” percept
7	Cacciaglia	2019	15	passive listenning + distracting movie	i) deviant > standard stimuli; ii) standard > deviant stimuli
8	Clos	2014	29	delayed matching to sample	Conjunction effects of i) match or ii) mismatch with prior information
9	Coste	2011	15	Stroop task	incongruent > baseline
10	Cross	2013	22	motion prediction	prediction > perception
11	Davis	2018	25	passive observation + button press	i) no regularity > regular location/category/both;
					ii) repetition effects in both category and location conditions
12	de Gardelle	2013	16	passive viewing + go/no go	i) alternated > repeated; ii) high repetition probability > low repetition probability
13	den Ouden	2009	16	target detection	four-way interaction reflecting sensitivity to surprising events (fit to a Rescorla-Wagner model)
14	den Ouden	2011	20	associative learning + gender discrimination	incongruent > congruent (modality aspecific)
15	Diekhof	2011	9	classification	misleading > correct expectations (imagery group)
16	Doricchi	2010	24	posner task	invalid > valid (when position is different from expectations)
17	Egner	2005	22	classification	incongruent-incongruent > congruent-incongruent
18	Eickhoff	2011	20	two-choice reaction time	correlation with response bias after probability change or stability
19	Esterman	2009	9	classification	expectation (preparatory activity before a stimulus arrives)
20	Fletcher	2001	11	passive viewing + go/no go	i) effect of initial learning ii) effect of umpredictable events
21	Friedman	2009	15	auditory oddball	novel > baseline
22	Gardner	2015	21	posture prediction or dot tracking	i) familiar > unfamiliar; ii) posture prediction > control attentional task
23	Geuter	2017	28	probabilistic heat pain	((cue high, warm) > (cue low, warm)) > ((cue high, pain) >(cue low, pain))

24	Grassi	2018	17	passive viewing + response press	alternative percept > default percept
25	Grotheer	2014	26	passive observation + response to stimuli	i) upright > inverted; ii) inverted > upright
26	Grotheer	2015	24	gender detection	i) alternate > repeated; ii) unexpected>expected repetitions
27	Hakonen	2017	20	passive listelling + judgement task	i) 2nd distorted > 1st distorted; ii) intact > 1st distorted
28	Harrison	2007	13	target identification	correlation with mutual information
29	Harrison	2007	12	attentional task	coherent >uncoherent motion
30	Hillebrandt	2013	14	social decision making	director present, 3 objects > 1 object
31	Johnson (Exp. A)	2016	16	observation + forced choice	random > patterned
31	Johnson (Exp. B)	2016	16	observation + forced choice	incongruent > congruent
32	Kandylaki	2016	20	passive listenning + comprehension task	i) passive > active verbs; ii) high causality > low causality
33	Kellerman	2017	35	following motion direction	arbitrary > random > predictable
34	Kluger	2017	20	detection of features	i) prediction errors > checkpoints (term > check ∩ ext > check);<br>ii) expectation compliance (term > ext); iii) parametric effect of surprise
35	Kronbichler	2018	36	passive viewing	i) repeated > new stimuli; ii) high probability> low probability
36	Langner	2011	24	go-no go	i) conjunction preparatory attention across modalities in cue only trials;<br>ii) cue only trials > predictive trials across modalities
37	Ligneul	2018	22	quiz	surprise recently experienced > surprise for each trial
38	Limanowski	2015	20	rubber hand illusion	congruent > incongruent (temporal integration)
39	Limongi	2016	16	visual prediction of motion	i) change > false alarm and no change; ii) time estimation error;<br>iii) no change > false alarm and change
40	Maffei (Exp. A)	2014	14	identification	backward > forward walking
40	Maffei (Exp. B)	2014	15	free viewing	backward > forward walking
41	Maffei	2015	15	alternative forced choice discrimination	i) intact > scrambled; ii) normal gravity > reduced gravity
42	Malekshahi	2016	12	continuous visual detection	deviant detection (explicit) > undetected deviants
43	Mayrhauser	2014	19	passive viewing + response press	repeated > alternated
44	McAndrews	2016	15	forced choice	i) incongruent 2 > incongruent 1; ii) congruent > incongruent
45	Megumi	2015	18	passive viewing + response when perceipt	rivalry-switch > replay-switch (spontaneous > stimulus-driven)
46	Nazimek	2013	12	associative learning	i) unexpected sound > expected sound;<br>ii) unexpected–expected sound > unexpected–expected silence

47	Noppeney	2007	17	forced choice task	incongruent vs. congruent (averaging sensory modality)
48	Okada	2018	20	tongue-twisters production + response	articulation > imagination (mismatch with motor plans)
49	Olkkonen	2017	12	passive viewing + go/no go	expectation effect (trial x block interaction)
50	Ondobaka	2014	15	movement and concept matching	i) congruency effect (movement/conceptual congruent > incongruent);
					ii) error effect (movement/conceptual congruent > incongruent)
51	O'Reilly	2013	17	perform saccades	Modelled effect of surprise; modelled effect of updating
52	Osnes	2012	19	passive listenning	incongruent > congruent music; vowel expectation
53	Parks	2012	28	guessing + reward	correlation with prediction error model parameter
54	Poppenk	2016	17	classification	i) novel > known or repeated proverbs; ii) known or repeated proverbs > novel
55	Rahnev	2011	21	viewing + forced choice task	predictive (valid + invalid) > nonpredictive (neutral)
56	Ran	2016	19	orientation identification	unpredictable > predictable
57	Rohe	2012	59	guessing with reward	reward prediction error > reward receipt
58	Saygin	2012	20	passive observation	repeated > non repeated
59	Schiffer	2012	19	passive viewing + multiple choice questions	i) solidity contrast (divergents with high or weak model solidity > repeated);
					ii) adaptation contrast (first > last)
60	Seymour	2005	19	visual distractors + classical pain conditioning	umpredicted stimuli > predicted
61	Soldan	2008	14	passive viewing + go/no go	repeated > non repeated
62	Thomas	2018	22	passive observation	intact > scrambled
63	Thompson	2008	16	passive viewing + go/no go	attended and unattended changes (change > repetition of color and shape)
64	Thornton	2019	28	social consequences rating	likely vs. unlikely future mental states; predictable vs. unpredictable sequences
65	Tipper	2015	46	passive observation + multiple choice	novel > repeated (dance and pantomime)
66	Tuennerhof	2016	20	target detection	i) umprimed > primed; ii) fine structure primed > fine structure umprimed
67	Vuilleumier	2005	10	passive viewing + go/no go	new > old (attended stream)
68	Wallentin	2015	49	Stroop task	i) incongruent > congruent; ii) low > high frequency
69	Weilnhammer	2017	20	forced three-options choice	transitions > baseline (+ correlation to prediction error model)
70	Yomogida	2010	24	videogame-like task	oddball > control

Table S2. Task modality and predictive phenomenon of fMRI experiments included in the meta-analysis.

ID	First author	Year	N	Modality	Stimuli	Predictive phenomeon reflected
1	Allen	2016	30	somatosensory	tactile/painful stimuli	Prediction Violation
2	Andics	2013	18	auditory	auditory words	Prediction Encoding
3	Barascud	2015	16	auditory	sequences of tones	Prediction Encoding
4	Bartels	2007	8	visual	natural scenes videos	Prediction Violation
5	Blank	2013	19	visual-auditory	faces/motion	Prediction Violation
6	Blank	2018	24	visual-auditory	words	Prediction Violation
7	Cacciaglia	2019	15	auditory	sounds	Prediction Violation and Encoding
8	Clos	2014	29	auditory	degraded or intact sentences	Prediction Violation and Encoding
9	Coste	2011	15	visual	colored words	Prediction Violation
10	Cross	2013	22	visual	moving agents	Prediction Encoding
11	Davis	2018	25	visual	faces, houses, flowers and tools	Prediction Violation and Encoding
12	de Gardelle	2013	16	visual	faces	Prediction Encoding
13	den Ouden	2009	16	visual-auditory	sounds or notes/visual shapes	Prediction Violation
14	den Ouden	2011	20	visual-auditory	sounds + faces	Prediction Violation
15	Diekhof	2011	9	visual	emotional faces	Prediction Violation
16	Doricchi	2010	24	visual	shapes and arrows	Prediction Violation
17	Egner	2005	22	visual	faces	Prediction Encoding
18	Eickhoff	2011	20	visual	arrows	Prediction Encoding
19	Esterman	2009	9	visual	faces and houses	Prediction Encoding
20	Fletcher	2001	11	visual	case studies + outcomes	Prediction Violation and Encoding
21	Friedman	2009	15	auditory	pure tones and encironmental sounds	Prediction Violation
22	Gardner	2015	21	visual	professional dancer, coloured dots	Prediction Encoding
23	Geuter	2017	28	visual-nociceptive	heat	Prediction Violation
24	Grassi	2018	17	visual	bistable shapes	Prediction Violation
25	Grotheer	2014	26	visual	inverted and upright faces	Prediction Violation and Encoding
26	Grotheer	2015	24	visual	faces photographs	Prediction Violation
27	Hakonen	2017	20	auditory	distorted sentences	Prediction Violation and Encoding
28	Harrison (Exp. A/B)	2007	13	visual	coloured shapes, moving shapes	Prediction Encoding
29	Harrison	2007	12	visual	Moving shapes	Prediction Encoding
30	Hillebrandt	2013	14	visual-auditory	visual social scenes + auditory instructions	Predition Encoding
31	Johnson (Exp. A/B)	2016	16	visual-auditory	videos + narration	Prediction Violation
32	Kandylaki	2016	20	auditory	stories	Prediction Violation and Encoding
33	Kellerman	2017	35	visual	white shapes	Prediction Violation
34	Kluger	2017	20	visual	colored numbers	Prediciton Violation and Encoding

35	Kronbichler	2018	36	visual	line drawings objects	Prediction Encoding
36	Langner	2011	24	visual-auditory-tactile	visual cues + stimuli in several modalites	Prediction Violation and Encoding
37	Ligneul	2018	22	visual	questions and answers about movies	Prediction Violation
38	Limanowski	2015	20	somatosensory	dummy arm + visuotactile stimulation	Prediction Violation
39	Limongi	2016	16	visual	moving shapes	Prediction Violation and Encoding
40	Maffei (Exp. A/B)	2014	14	visual	people walking on- or backwards	Prediction Violation
41	Maffei	2015	15	visual	moving stick-figures	Prediction Encoding
42	Malekshahi	2016	12	visual	moving shapes	Prediction Violation
43	Mayrhauser	2014	19	visual	line-drawing of objects	Prediction Encoding
44	McAndrews	2016	15	visual	pictures	Prediction Encoding
45	Megumi	2015	18	visual	structure-from-motion shapes	Prediction Encoding
46	Nazimek	2013	12	auditory	sounds + visual shapes	Prediction Violation
47	Noppeney	2007	17	visual-auditory	picture and spoken words	Prediction Violation
48	Okada	2018	20	auditory	tongue twisters	Prediction Violation
49	Olkkonen	2017	12	visual	artificially generated faces	Prediction Encoding
50	Ondobaka	2014	15	visual-auditory	moving people and verbal cues	Prediction Violation and Encoding
51	O'Reilly	2013	17	visual	moving dots	Prediction Violation and Encoding
52	Osnes	2012	19	auditory	musical sounds and vowels	Prediction Violation
53	Parks	2012	28	visual	reward values and probability cues	Prediction Violation
54	Poppenk	2016	17	visual	proverbs	Prediction Violation and Encoding
55	Rahnev	2011	21	visual	moving dots	Prediction Encoding
56	Ran	2016	19	visual	words, faces, grating	Prediction Violation
57	Rohe	2012	59	visual	numbers	Prediction Violation
58	Saygin	2012	20	visual	humans/android/robot moving	Prediction Encoding
59	Schiffer	2012	19	visual	video of actions	Prediction Violation and Encoding
60	Seymour	2005	19	nociceptive	heat or cold	Prediction Violation
61	Soldan	2008	14	visual	real and unreal objects	Prediction Encoding
62	Thomas	2018	22	visual	human bodies/motion	Prediction Encoding
63	Thompson	2008	16	visual	shapes	Prediction Violation
64	Thornton	2019	28	visual	terms of inner states + social scenes	Prediction Encoding
65	Tipper	2015	46	visual	dance or pantomime videos	Prediction Encoding
66	Tuennerhof	2016	20	auditory	sentences	Prediction Violation and Encoding
67	Vuilleumier	2005	10	visual	shapes	Prediction Encoding
68	Wallentin	2015	49	visual-auditory	colored words	Prediction Violation
79	Weilnhammer	2017	20	visual	lissajous figures	Prediction Violation
70	Yomogida	2010	24	visual-auditory	moving shapes and auditory feedback	Prediction Violation

Table S3. Seed-voxel correlations (SVC) consensus results.

Condition	MNI coordinates (x,y,z)	Anatomical location (Brodmann area)
**General Prediction negative correlations**		
	18,-100,-22	Right uvula (cerebellum)
	-6,-36,84	Left precentral gyrus (BA 4)
	-28,-30,64	Left postcentral gyrus (BA 3)
	-42,-30,72	Left postcentral gyrus (BA 2)
	-58,-16,60	Left postcentral gyrus (BA 3)
	24,-76,4	Right middle occipital gyrus (BA 19)
	58,-14,60	Right postcentral gyrus (BA 1)
**General Prediction positive correlations**		
	-28,35,-18	Left inferior frontal gyrus (BA 47)
	-45,-28,3	Left superior temporal gyrus (BA 41)
	51,-16,-1	Right superior temporal gyrus (BA 22)
	-9,-7,14,1	Left thalamus
	-16,-57,-50	Left tonsil (cerebellum)
	-23-38,7	Left parahippocampus (BA 30)
	-23,-40,5	Left parahippocampus (BA 30)
**Prediction Violation negative correlations**		
	14,70,-10	Right medial frontal gyrus (BA 10)
	-2,-36,78	Left postcentral gyrus (BA 5)
	-56,-18,62	Left postcentral gyrus (BA 1)
	0,-6,22	Left cingulate gyrus (BA 24)
	58,-14,60	Right postcentral gyrus (BA 1)
	34,-60,74	Right superior parietal lobule (BA 7)
	-28,-30,66	Left postcentral gyrus (BA 2)
	24,-76,4	Right lingual gyrus (BA 18)
**Prediction Violation positive correlations**		
	-36,0,44	Left middle frontal gyrus (BA 6)
	-54,-40,-30	Left culmen (cerebellum)
	24,48,-6	Right anterior cingulate (BA 32)
	-16,32,-38	Left orbital gyrus (BA 47)
	-62,-72,-12	Left middle occipital gyrus (BA 18)
	-64,-68,-14	Left declive (cerebellum)
**Prediction Encoding negative correlations**		
	26,-98,-24	Right uvula (cerebellum)
	4,-48,-6	Right culmen (cerebellum)
	-6,-54,82	Left postcentral gyrus (BA 7)
	0,-18,38	Left cingulate gyrus (BA 24)
	20,-14,30	Right caudate body
	-20,-20,-14	Left parahippocampal gyrus (BA 35)

**Prediction Encoding positive correlations**		
	42,14,22	Right inferior frontal gyrus (BA 9)
	-24,-44,42	Left cingulate gyrus (BA 31)
	34,-48,50	Right superior parietal lobule (BA 7)
	52,-44,-14	Right fusiform gyrus (BA 37)
	8,16,54	Right superior frontal gyrus (BA 6)
	12,-74,-42	Right inferior semi-lunar lobe (cerebellum)
	-34,-2,-4	Left claustrum
	62,-36,16	Right insula (BA 13)

Table S4. Two-sample t-test comparing the SVC Consensus maps in the conditions of Prediction Encoding and Prediciton Violation
Threshold set at p <0.005, family-wise error (FWE) corrected. For a visual plot, see Figure S3.

**Condition**	**MNI coordinates (x,y,z)**	**Anatomical location (Brodmann area)**
**Prediction Violation > Prediction Encoding**		
	-4,6,8	Left frontal opercular area (BA 44)
	-20,38,24	Left medial frontal gyrus (BA 9)
	-12,22,24	Left anterior cingulate gyrus (BA 32)
	-14,10,70	Left medial frontal gyrus (BA 6)
	0,-20,-2	Red Nucleus
	44,32,-6	Right inferior frontal gyrus (BA 45)
	-40,4,52	Left middle frontal gyrus (BA 6)
	-60,-46,24	Left inferior parietal lobule (BA 40)
	32,10,-2	Right Claustrum
**Prediction Encoding > Prediction Violation** (Clusters > 5 voxels)		
	12,72,-8	Right prefrontal cortex (BA 10)
	6,-6,28	Right enthorinal cortex (BA 28)
	12,24,-34	Right rectal gyrus (BA 11)
	20,46,-26	Right middle frontal gyrus (BA 11)
	-26,-42,16	Left caudate tail
	-10,-2,-26	Left parahippocampus (BA 34)
	2,-26,-56	Right paracentral lobule (BA 5)
	-18,46,-26	Left superior frontal gyrus (BA 11)

Table S5. Number and proportion of experiments per sensory modality in each condition. Note that the number of experiments is higher than that of the studies.

Condition	Sensory modality	Number (percentage%) of articles
**General Prediction (total experiments = 73)**		
	Visual	43 (58,9 %)
	Audio-visual	10 (13,7 %)
	Auditory	11 (15,1 %)
	Somatosensory, tactile, interoceptive and mixed	5 (6,8 %)
**Prediction Violation (total experiments = 46)**		
	Visual	23 (50,0 %)
	Audio-visual	10 (21,7 %)
	Auditory	8 (17,4 %)
	Somatosensory, tactile, interoceptive and mixed	5 (10,9 %)
**Prediction Encoding (total experiments = 39)**		
	Visual	29 (74,4 %)
	Audio-visual	3 (7,7 %)
	Auditory	6 (15,4 %)
	Somatosensory, tactile, interoceptive and mixed	1 (2,6 %)

Figure S1. Overlap between the SVC Consensus maps calculated with and without voxels located around the seed in the SVC maps (local connectivity).

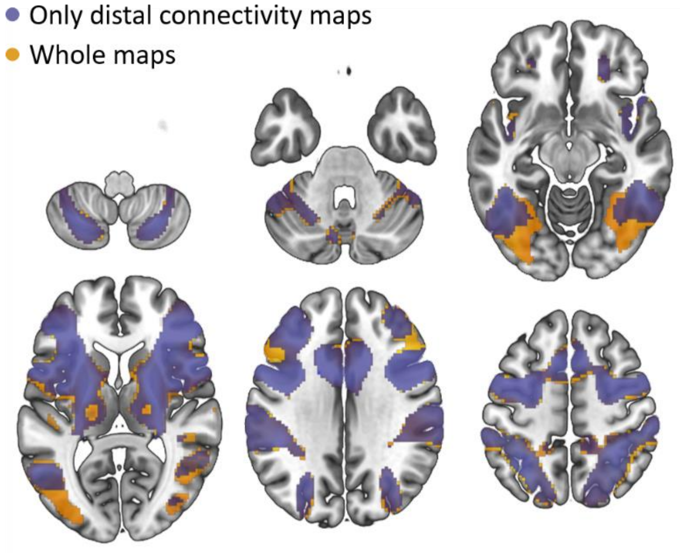

Figure S1: Overlap between the SVC consensus map for the condition of General Prediction considering all the voxels (yellow) and the SVC consensus calculated excluding the local connectivity (blue), i.e. the voxel located within 14 mm³ from the seed when calculating the original SVC maps.

Figure S2. SVC Consensus results for Prediction Violation and Prediction Encoding conditions.

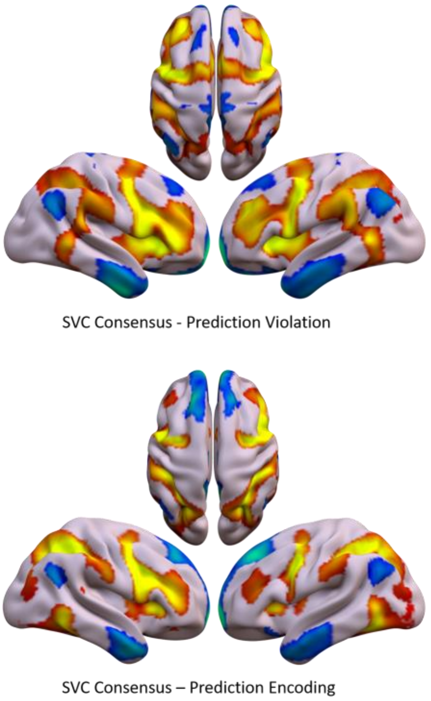

Figure S2: Surface, medial and cerebellar mapping of the SVC consensus analysis revealing the "predictive network" for the conditions of Prediction Violation (upper panel) and Prediction Encoding (lower panel). Warm colors indicate positive t-values (Prediction Violation range: 4.4-11.9; Prediction Encoding range: 4.4-8.8), cold colors indicate negative t-values (Prediction Violation range: 4.4-17.9; Prediction Encoding range: 4.4-12.5). Shown in arbitrary units.

Figure S3. Two-sample t-test results comparing the maps of the Predition Violation and the Prediction Encoding condition

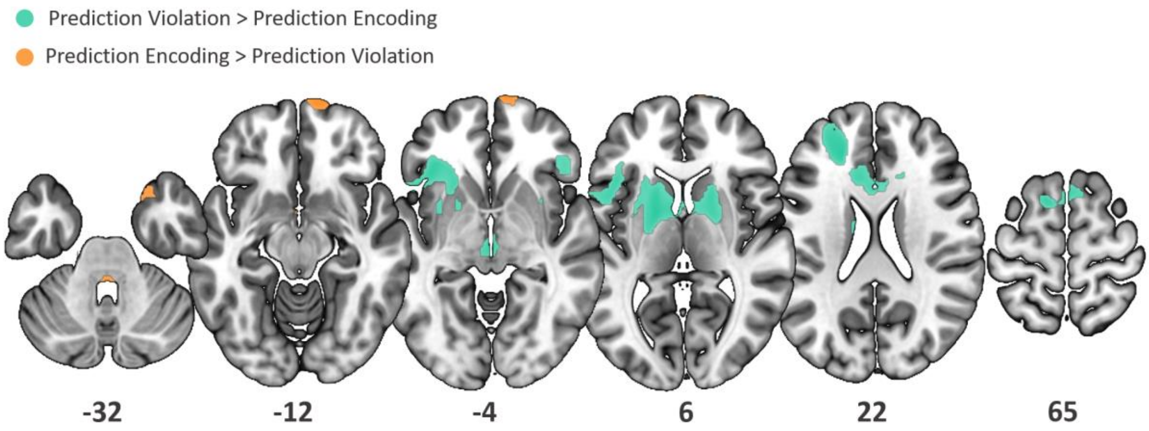

Figure S3: Results of the two-sample t-test comparing the SVC Consensus maps of Prediciton Violation and Prediction Encoding. The contrast Prediction Violation > Prediction Encoding is in green, while the contrast Prediction Encoding > Prediction Violation is in orange. Results are thresholded at p<0.005, with a FWE correction (for further details, see Table S4).

Supplementary References. Original studies included in the meta-analysis.

1. Allen M, Fardo F, Dietz MJ, Hillebrandt H, Friston KJ, Rees G, et al. Anterior insula coordinates hierarchical processing of tactile mismatch responses. NeuroImage. 2016;127:34-43.

2. Andics A, Gál V, Vicsi K, Rudas G, Vidnyánszky Z. FMRI repetition suppression for voices is modulated by stimulus expectations. NeuroImage. 2013;69:277-83.

3. Balser N, Lorey B, Pilgramm S, Naumann T, Kindermann S, Stark R, et al. The influence of expertise on brain activation of the action observation network during anticipation of tennis and volleyball serves. Frontiers in human neuroscience. 2014;8:568.

4. Barascud N, Pearce MT, Griffiths TD, Friston KJ, Chait M. Brain responses in humans reveal ideal observer-like sensitivity to complex acoustic patterns. Proceedings of the National Academy of Sciences of the United States of America. 2016;113(5):E616-25.

5. Bartels A, Zeki S, Logothetis NK. Natural vision reveals regional specialization to local motion and to contrast-invariant, global flow in the human brain. Cerebral cortex. 2008;18(3):705-17.

6. Blank H, von Kriegstein K. Mechanisms of enhancing visual–speech recognition by prior auditory information. NeuroImage. 2013;65:109-18.

7. Blank H, Spangenberg M, Davis MH. Neural prediction errors distinguish perception and misperception of speech. Journal of Neuroscience. 2018;38(27):6076-89.

8. Cacciaglia R, Costa-Faidella J, Zarnowiec K, Grimm S, Escera C. Auditory predictions shape the neural responses to stimulus repetition and sensory change. NeuroImage. 2019;186:200-10.

9. Clos M, Langner R, Meyer M, Oechslin MS, Zilles K, Eickhoff SB. Effects of prior information on decoding degraded speech: an fMRI study. Human brain mapping. 2014;35(1):61-74.

10. Coste C, Sadaghiani S., Friston K., & Kleinschmidt A., Ongoing Brain Activity Fluctuations Directly Account for Intertrial and Indirectly for Intersubject Variability in Stroop Task Performance. Cerebral Cortex. 2011; 21, 2612-2619

11. Cross ES, Stadler W, Parkinson J, Schütz-Bosbach S, Prinz W. The influence of visual training on predicting complex action sequences. Human brain mapping. 2013;34(2):467-86.

12. Davis B, Hasson U. Predictability of what or where reduces brain activity, but a bottleneck occurs when both are predictable. NeuroImage. 2018;167:224-36.

13. Den Ouden HE, Friston KJ, Daw ND, McIntosh AR, Stephan KE. A dual role for prediction error in associative learning. Cerebral cortex. 2009;19(5):1175-85.

14. den Ouden HE, Daunizeau J, Roiser J, Friston KJ, Stephan KE. Striatal prediction error modulates cortical coupling. Journal of Neuroscience. 2010;30(9):3210-9.

15. Diekhof EK, Kipshagen HE, Falkai P, Dechent P, Baudewig J, Gruber O. The power of imagination—How anticipatory mental imagery alters perceptual processing of fearful facial expressions. NeuroImage. 2011;54(2):1703-14.

16. Doricchi F, Macci E, Silvetti M, Macaluso E. Neural correlates of the spatial and expectancy components of endogenous and stimulus-driven orienting of attention in the Posner task. Cerebral Cortex. 2010;20(7):1574-85.

17. Egner T, Hirsch J. Cognitive control mechanisms resolve conflict through cortical amplification of task-relevant information. Nature neuroscience. 2005;8(12):1784-90.

18. Eickhoff SB, Pomjanski W, Jakobs O, Zilles K, Langner R. Neural correlates of developing and adapting behavioral biases in speeded choice reactions—an fMRI study on predictive motor coding. Cerebral cortex. 2011;21(5):1178-91.

19. Esterman M, Yantis S. Perceptual expectation evokes category-selective cortical activity. Cerebral Cortex. 2010;20(5):1245-53.

20. Fletcher PC, Anderson J, Shanks D, Honey R, Carpenter TA, Donovan T, et al. Responses of human frontal cortex to surprising events are predicted by formal associative learning theory. Nature neuroscience. 2001;4(10):1043-8.

21. Friedman D, Goldman R, Stern Y, Brown TR. The brain's orienting response: An event-related functional magnetic resonance imaging investigation. Human brain mapping. 2009;30(4):1144-54.

22. Gardner T, Goulden N, Cross ES. Dynamic modulation of the action observation network by movement familiarity. Journal of Neuroscience. 2015;35(4):1561-72.

23. Geuter S, Boll S, Eippert F, Büchel C. Functional dissociation of stimulus intensity encoding and predictive coding of pain in the insula. eLife. 2017;6:e24770.

24. Grassi PR, Zaretskaya N, Bartels A. A generic mechanism for perceptual organization in the parietal cortex. Journal of Neuroscience. 2018;38(32):7158-69.

25. Grotheer M, Hermann P, Vidnyánszky Z, Kovács G. Repetition probability effects for inverted faces. NeuroImage. 2014;102 Pt 2:416-23.

26. Grotheer M, Kovács G. The relationship between stimulus repetitions and fulfilled expectations. Neuropsychologia. 2015;67:175-82.

27. Hakonen M, May PJC, Jääskeläinen IP, Jokinen E, Sams M, Tiitinen H. Predictive processing increases intelligibility of acoustically distorted speech: Behavioral and neural correlates. Brain and behavior. 2017;7(9):e00789.

28. Harrison LM, Duggins A, Friston KJ. Encoding uncertainty in the hippocampus. Neural Networks. 2006;19(5):535-46.

29. Harrison LM, Stephan KE, Rees G, Friston KJ. Extra-classical receptive field effects measured in striate cortex with fMRI. NeuroImage. 2007;34(3):1199-208.

30. Hillebrandt H, Dumontheil I, Blakemore S-J, Roiser JP. Dynamic causal modelling of effective connectivity during perspective taking in a communicative task. NeuroImage. 2013;76:116-24.

31. Johnson MA, Turk-Browne NB, Goldberg AE. Neural systems involved in processing novel linguistic constructions and their visual referents. Language, cognition and neuroscience. 2016;31(1):129-44.

32. Kandylaki KD, Nagels A, Tune S, Kircher T, Wiese R, Schlesewsky M, et al. Predicting “when” in discourse engages the human dorsal auditory stream: An fMRI study using naturalistic stories. Journal of Neuroscience. 2016;36(48):12180-91.

33. Kellermann T, Scholle R, Schneider F, Habel U. Decreasing predictability of visual motion enhances feed-forward processing in visual cortex when stimuli are behaviorally relevant. Brain Structure and Function. 2017;222(2):849-66.

34. Kluger DS, Schubotz RI. Strategic adaptation to non-reward prediction error qualities and irreducible uncertainty in fMRI. Cortex; a journal devoted to the study of the nervous system and behavior. 2017;97:32-48.

35. Kronbichler L, Said-Yürekli S, Kronbichler M. Perceptual expectations of object stimuli modulate repetition suppression in a delayed repetition design. Scientific reports. 2018;8(1):1-8.

36. Langner R, Kellermann T, Boers F, Sturm W, Willmes K, Eickhoff SB. Modality-specific perceptual expectations selectively modulate baseline activity in auditory, somatosensory, and visual cortices. Cerebral cortex. 2011;21(12):2850-62.

37. Ligneul R, Mermillod M, Morisseau T. From relief to surprise: Dual control of epistemic curiosity in the human brain. NeuroImage. 2018;181:490-500.

38. Limanowski J, Blankenburg F. Network activity underlying the illusory self-attribution of a dummy arm. Human brain mapping. 2015;36(6):2284-304.

39. Limongi R, Pérez FJ, Modroño C, González-Mora JL. Temporal Uncertainty and Temporal Estimation Errors Affect Insular Activity and the Frontostriatal Indirect Pathway during Action Update: A Predictive Coding Study. Frontiers in human neuroscience. 2016;10(276).

40. Maffei V, Giusti MA, Macaluso E, Lacquaniti F, Viviani P. Unfamiliar Walking Movements Are Detected Early in the Visual Stream: An fMRI Study. Cerebral cortex (New York, NY : 1991). 2015;25(8):2022-34.

41. Maffei V, Indovina I, Macaluso E, Ivanenko YP, G AO, Lacquaniti F. Visual gravity cues in the interpretation of biological movements: neural correlates in humans. NeuroImage. 2015;104:221-30.

42. Malekshahi R, Seth A, Papanikolaou A, Mathews Z, Birbaumer N, Verschure PF, et al. Differential neural mechanisms for early and late prediction error detection. Scientific reports. 2016;6:24350.

43. Mayrhauser L, Bergmann J, Crone J, Kronbichler M. Neural repetition suppression: evidence for perceptual expectation in object-selective regions. Frontiers in human neuroscience. 2014;8(225).

44. McAndrews MP, Girard TA, Wilkins LK, McCormick C. Semantic congruence affects hippocampal response to repetition of visual associations. Neuropsychologia. 2016;90:235-42.

45. Megumi F, Bahrami B, Kanai R, Rees G. Brain activity dynamics in human parietal regions during spontaneous switches in bistable perception. NeuroImage. 2015;107:190-7.

46. Michelon P, Snyder AZ, Buckner RL, McAvoy M, Zacks JM. Neural correlates of incongruous visual information. An event-related fMRI study. NeuroImage. 2003;19(4):1612-26.

47. Nazimek JM, Hunter MD, Hoskin R, Wilkinson I, Woodruff PW. Neural basis of auditory expectation within temporal cortex. Neuropsychologia. 2013;51(11):2245-50.

48. Noppeney U, Josephs O, Hocking J, Price CJ, Friston KJ. The effect of prior visual information on recognition of speech and sounds. Cerebral cortex (New York, NY : 1991). 2008;18(3):598-609.

49. O'Reilly JX, Schüffelgen U, Cuell SF, Behrens TE, Mars RB, Rushworth MF. Dissociable effects of surprise and model update in parietal and anterior cingulate cortex. Proceedings of the National Academy of Sciences of the United States of America. 2013;110(38):E3660-9.

50. Okada K, Matchin W, Hickok G. Neural evidence for predictive coding in auditory cortex during speech production. Psychonomic bulletin & review. 2018;25(1):423-30.

51. Olkkonen M, Aguirre GK, Epstein RA. Expectation modulates repetition priming under high stimulus variability. Journal of vision. 2017;17(6):10.

52. Ondobaka S, de Lange FP, Wittmann M, Frith CD, Bekkering H. Interplay Between Conceptual Expectations and Movement Predictions Underlies Action Understanding. Cerebral cortex (New York, NY : 1991). 2015;25(9):2566-73.

53. Rahnev D, Lau H, de Lange FP. Prior expectation modulates the interaction between sensory and prefrontal regions in the human brain. The Journal of neuroscience : the official journal of the Society for Neuroscience. 2011;31(29):10741-8.

54. Ran G, Chen X, Cao X, Zhang Q. Prediction and unconscious attention operate synergistically to facilitate stimulus processing: An fMRI study. Consciousness and cognition. 2016;44:41-50.

55. Rohe T, Weber B, Fliessbach K. Dissociation of BOLD responses to reward prediction errors and reward receipt by a model comparison. The European journal of neuroscience. 2012;36(3):2376-82.

56. Saygin AP, Chaminade T, Ishiguro H, Driver J, Frith C. The thing that should not be: predictive coding and the uncanny valley in perceiving human and humanoid robot actions. Social cognitive and affective neuroscience. 2012;7(4):413-22.

57. Schiffer AM, Ahlheim C, Ulrichs K, Schubotz RI. Neural changes when actions change: adaptation of strong and weak expectations. Human brain mapping. 2013;34(7):1713-27.

58. Seymour B, O'Doherty JP, Koltzenburg M, Wiech K, Frackowiak R, Friston K, et al. Opponent appetitive-aversive neural processes underlie predictive learning of pain relief. Nature neuroscience. 2005;8(9):1234-40.

59. Soldan A, Zarahn E, Hilton HJ, Stern Y. Global familiarity of visual stimuli affects repetition-related neural plasticity but not repetition priming. NeuroImage. 2008;39(1):515-26.

60. Thomas RM, De Sanctis T, Gazzola V, Keysers C. Where and how our brain represents the temporal structure of observed action. NeuroImage. 2018;183:677-97.

61. Thompson R, Duncan J. Attentional modulation of stimulus representation in human fronto-parietal cortex. NeuroImage. 2009;48(2):436-48.

62. Thornton MA, Weaverdyck ME, Tamir DI. The Social Brain Automatically Predicts Others' Future Mental States. The Journal of neuroscience : the official journal of the Society for Neuroscience. 2019;39(1):140-8.

63. Tipper CM, Signorini G, Grafton ST. Body language in the brain: constructing meaning from expressive movement. Frontiers in human neuroscience. 2015;9:450.

64. Tuennerhoff J, Noppeney U. When sentences live up to your expectations. NeuroImage. 2016;124(Pt A):641-53.

65. Vuilleumier P, Schwartz S, Duhoux S, Dolan RJ, Driver J. Selective attention modulates neural substrates of repetition priming and "implicit" visual memory: suppressions and enhancements revealed by FMRI. Journal of cognitive neuroscience. 2005;17(8):1245-60.

66. Wallentin M, Gravholt CH, Skakkebæk A. Broca's region and Visual Word Form Area activation differ during a predictive Stroop task. Cortex; a journal devoted to the study of the nervous system and behavior. 2015;73:257-70.

67. Weber K, Lau EF, Stillerman B, Kuperberg GR. The Yin and the Yang of Prediction: An fMRI Study of Semantic Predictive Processing. PloS one. 2016;11(3):e0148637.

68. Weilnhammer V, Stuke H, Hesselmann G, Sterzer P, Schmack K. A predictive coding account of bistable perception - a model-based fMRI study. PLoS computational biology. 2017;13(5):e1005536.

69. Yomogida Y, Sugiura M, Sassa Y, Wakusawa K, Sekiguchi A, Fukushima A, et al. The neural basis of agency: an fMRI study. NeuroImage. 2010;50(1):198-207.
